# Experimental Medicine

**Published:** 1868-04-15

**Authors:** L. Ranvier

**Affiliations:** Paris


### CHICAGO MEDICAL JOURNAL.

*Vol. XXV.*—*APRIL* 15, 1868.—*No.* 8.

### EXPERIMENTAL MEDICINE.

*Experimental Researches into the Subject of the Action of Phosphorus upon Living Tissues.—Reflections upon the Pathogenesis of Fatty Transformations. By Dr. L. Ranvier, Paris.*

TRANSLATED EXPRESSLY FOR THE JOURNAL, BY WALTER HAY, M.D., CHICAGO.

Fatty transformations are so common, and are surrounded by so great obscurity in their pathogenesis, that the means for producing them experimentally become necessarily very interesting. Since it became known that *Phosphorus*, introduced into the economy, determined, in the liver, the kidneys, the muscles, etc., fatty transformations, complete and very rapid, numerous investigations have been undertaken upon poisoning by this substance.

Since the memoir which we published in 1863,* in which is published a complete history of the question up to this epoch, new observations have been published. Several have chosen this subject for their inaugural theses; certain professors have

* Fritz, Ranvier et Verliae. De la Stéatose dans L'Empoisonnement par le Phosphore. Arch. Gen. de Méd., 1863.

treated it in their instructions; * but no new opinion has been advanced in France touching the pathogenesis of these fatty transformations.

The same can not be said of Germany, where several (three) authors have occupied themselves with the mode of action of *Phosphorus* upon the tissues, in producing in them fatty transformations. Only, in place of seeking to clear up, by means of experiment, the obscurity which rests upon the subject of fatty transformations in general, they have made experiments, with a preconceived theory, upon the pathogenesis of fatty transformations. This theory originated in Berlin. Indeed, after having studied the inflammatory lesions of some organs (the liver, kidneys, and muscles,) Virchow classifies these lesions, according as they involve the connective stroma of these organs, or their special histological elements.

In the first case, the predominant phenomenon is a multiplication of the cellular elements of the connective tissue of the stroma; and the inflammation characterized by this phenomenon receives the name of *Interstitial.*

In the second case, the special histological elements, hepatic cells, epithelium of tubuli uriniferi, and striated fasciculi, after having presented some characteristics of proliferation, and having been infiltrated with an albuminoid substance, undergo fatty transformations. The inflammation manifesting itself essentially by this phenomenon, has been designated under the name of parenchymatous; and the fatty transformation

* Lancereaux, Etude sur la Déginérescence Graissense des Elements Actifs du Foie, des Reins, et des Muscles de la vie Animale dans L'Empoisonnement par le Phosphore, Union Méd., 1863.

L. Branet. De L'Empoisonnement Aiga par le Phosphore, th., 1863.

E. Fabre. De la Dégénérescence Graissense dans L'Empoisonnement Aiga par le Phosphore, th., 1864.

Cornil et Bergeron. Altération Granulo-Graissense de L'Epithélium des Glandes de L'Estomæ dans un Cas de L'Empoissonnement par le Phosphore. (Soc. de Biol., 1865.)

Tardein. Leçons sur L'Empoisonnement par le Phosphore. (Gaz. des Hôp., 1864.)

Blachez, la Stéatose, th. agr., 1866.

becomes the most important sign of this sort of inflammation. If it has not always been taught in the school, that the fatty transformation of the muscles and of the tubuli uriniferi, for example, manifests always inflammation, at least one is induced to suppose an inflammatory origin to these alterations, in the absence of every other cause.

Moreover, a pupil of this school, Dr. Mannkopff,* maintains that in poisoning by *Phosphorus*, the fatty transformations depend upon an inflammation determined by the irritant action of the toxic matter. He relied especially upon the examination of the liver and kidneys, which, according to his observations, should present, at the same time with the steatosis, hypertrophy of their stroma.

Since I became acquainted with the opinion of this author, I have examined, with the greatest care, subjects poisoned by *Phosphorus*, and animals which I have subjected to intoxication by this substance, to determine whether the stroma of the kidney and of the liver presented any hyperplastic thickening; and I must say that my researches into this subject have always met with negative results. I believe that some error has crept into the observations of Dr. Mannkopff: close attention is necessary, and also excellent lenses, in order to appreciate slight hyperplasias of conjunctive tissues which occupy the spaces left between the canaliculi uriniferi and the hepatic cells; for these spaces are traversed by vessels whose nuclei and sanguineous globules can, after hardening in chromic acid, deceive by their size, insufficient for corpuscles of connective tissue.

Virchow † himself maintains the inflammatory nature of

* Mannkopff, Beitrag zur Lehre von der Phosphore Vergiftung. (Wein Méd. Wochenseh, 1863.)

Barjau. Wiener Zeitschr. der Aerzto., 1863.

† Tiingel. Arch. de Virchow, 1864.

Virchow. *Id.*, 1864.

Ludwig Meyer. *Id.*, 1865.

Klebs. *Id.*, 1865.

Pastau. *Id.*, 1865.

Vohl. Berl. Klin. Wocheusch, 1865.

fatty transformations, determined by *Phosphorus;* for, having discovered in the stomachs of subjects dead from poisoning, a fatty transformation of the pepsine glands, he considered it the result of the irritant action of *Phosphorus*, and designated it gastro-adenitis. This mode of opinion has been sanctioned in Germany; and in a recent work, Dr. Hugo Senftleben * admits it without any discussion. It is, however, very open to discussion.

First, then, it is known that *Phosphorus* ingested does not always determine inflammatory lesions of the gastro-intestinal mucous membrane; for, in a number of autopsies, there has not been found, in the stomach or in the intestines, the least inflammatory lesion. In some cases, however, hyperæmias, and even ulcerations, have been observed. I have myself met with ulcerations of the gastric mucous membrane, in a dog to which I had administered *Phosphorus* in solution in *Sulphuric ether*.

In order to produce hyperæmia of the gastric mucous membrane, it is not necessary to administer *Phosphorus* by the digestive canal; for Dr. Senftleben states that he has observed congestion of the duodenum and stomach, in animals that had died in consequence of injections of *Phosphoretted oil* into the cellular tissue. In these cases, the stomach and intestine contain an unctuous substance of a grayish color, formed of epithelial cells, filled with fatty granulations.

That similar lesions exist in the digestive canal, does not follow solely as a direct result of the action of *Phosphorus;* for these lesions could very well depend upon the corrosive action of the gastric juice upon the mucous membrane, denuded of its epithelium. Indeed, does not the fatty degeneration of some of the glands of the stomach and of the epithelial investment of the gastro-intestinal mucous membrane, leave the denuded surface without defence against the irritant action of the gastric and intestinal juices? It is well known that embolias of the capillaries of the digestive mucus membrane, and pemphigus of this surface, give origin to ulcerations of rapid

* Hugo Senftleben. Arch. de Virchow, 1866.

progress, explained by the presence of the gastric juice. I have met, in the case of an old man, with a fatty transformation of a portion of the digestive mucous surface, which had occasioned ulcerations accompanied by hæmatemesis.* It is clear that in these different cases, the epithelial investment no longer exists; the mucous chorion, being no longer protected, has been attacked by the gastric juice.

Moreover, do not the experiments of Senftleben combat the idea of gastritis by the irritant action of *Phosphorus*, since, in these experiments, the toxic element has not been placed in contact with the gastro-intestinal mucous membrane? It seems logical, then, to admit that the fatty *dêbris* found in the digestive canal, consists of degenerated and desquamated epithelium; and that the redness of the mucous membrane results from the irritant action of the juices upon the denuded surface.

In some case, moreover, it has been determined that the mucous surface has presented neither redness nor ulceration; although the glands had undergone fatty transformation. It might be that in these cases the alteration was in its incipiency, or that all the glands, having undergone degeneration at the same time, could not any longer secrete the corrosive juice. This is, however, merely an hypothesis which may be hereafter verified or falsified by facts.

I come now to the experimental portion of this work.

In order to form an opinion touching the irritant action of *Phosphorus* upon living tissues, it suffices to place some fragments of this substance under the skin and between the muscles of different animals, and to observe whether they determine there the phenomena of inflammation. My experiments have been made upon the frog, the guinea-pig, and the rabbit. I have proceeded in the following manner: I cut from a stick of *Phosphorus*, a small, regular cube, taking care that there remained upon its surface no portion of the coating of white dust, which always covers *Phosphorus* preserved in water, exposed to the light. I measured this little cube, thus

* Bull. de la Soc. Anato., 1863.

obtained, and slipped it through a sub-cutaneous incision into the locality where I desired it to remain.

### FIRST SERIES OF EXPERIMENTS.

On the 13th of September, I took three frogs, equally large and vigorous. I cut from a stick of *Phosphorus*, three cubes, having sides of one millimetre in length. These fragments were placed, one between the posterior muscles of the leg of one of the frogs, another under the skin of the loins of another frog, and the last was pushed into the œsophagus of the third frog. These animals were placed in different vessels, and placed in a cool situation. Other healthy frogs were preserved under similar conditions.

During the following days, up to the 30th of September, these different animals presented nothing worthy of note.

*Experiment* 1.— The first of October, I watched the death-struggle of the first frog. The animal was in a state of muscular relaxation, interrupted, from time to time, by certain slight convulsive movements. Death supervened an hour after the beginning of my observation.

The fragment of *Phosphorus* remained in the position where I had placed it. It had preserved its transparency; its angles remained sharp. I could not establish any very perceptible diminution in its dimensions; from which it is necessary to conclude that the quantity of matter absorbed had been very small. The muscles of the leg in the midst of which the *Phosphorus* was found, presented no swelling or redness. Under the microscope, the nuclei were not augmented in number; and their primitive fasciculi were spangled with fine fatty granulations, occupying the depressions left between the elementary fibrillæ.

The presence of fatty granulations in the muscular fasciculi in the neighborhood of the piece of *Phosphorus*, could have been attributed to a toxic action, either local or general. To determine this, I examined the other muscles of the same animal, and found in them fatty granulations, in number just as considerable.

The fatty transformation of the muscles, then, could not, therefore, be attributed to the local action of the *Phosphorus*. In order to judge the second part of the question, I examined the muscles of a healthy frog; and I observed that all the muscular fasciculi of this frog contained fatty granulations. I continued this investigation in all the frogs which I could procure at that time, and I observed that all of them had granulo-fatty muscles.

When, therefore, any one may make, in the autumn, experiments upon frogs, having for their object the determination amongst them of fatty transformations, it is necessary to be acquainted with this physiological condition of the muscles. Recently, there has appeared in the "Journal" of M. Schulze, a work, apparently detailed by A. Stuart, upon the fatty transformations of muscles supervening upon the application of irritating substances. Almost all the experiments of this author have been conducted upon the frog; and, moreover, he seemed to be ignorant that in autumn these animals have their muscles loaded with fatty granulations.

The liver, the kidneys and the heart of the frog which we examined at that time, presented, to the naked eye and to the microscope, the characteristics of complete fatty degeneration. The liver was of a yellow color, its volume was nearly doubled, its edges softened; its cells not destroyed at any point, but loaded with little drops, and with fine fatty granulations. The kidneys were furrowed with yellowish stripes, and the epithelial cells of their tubeli were infiltrated with fat.

*Experiment* 2. — The second frog died on the 8th of October, twenty-five days after the introduction of *Phosphorus* under the skin of the lumbar region. I discovered the fragment in the position where I had placed it. It had undergone no apparent alteration, nor had it determined any inflammation in its neighborhood; not the least adhesion, no redness, no serosity. The muscles presented the same characteristics as those of the preceding frog. The viscera were in a state of advanced fatty degeneration.

*Experiment* 3. — The third frog died the next day, the 9th of October, twenty-six days after the introduction of the *Phosphorus* into the digestive canal. I did not discover the fragment, either in the stomach or in the intestine. It was probably rejected. I did not determine any alteration of the gastro-intestinal mucous membrane. The muscles were granulo-fatty; the kidneys and liver were completely degenerated.

(*To be continued.*)

